# Postoperative Cavity Stereotactic Radiosurgery for Brain Metastases

**DOI:** 10.3389/fonc.2018.00342

**Published:** 2018-08-31

**Authors:** Eduardo M. Marchan, Jennifer Peterson, Terence T. Sio, Kaisorn L. Chaichana, Anna C. Harrell, Henry Ruiz-Garcia, Anita Mahajan, Paul D. Brown, Daniel M. Trifiletti

**Affiliations:** ^1^Department of Radiation Oncology, Mayo Clinic, Jacksonville, FL, United States; ^2^Department of Radiation Oncology, Mayo Clinic, Phoenix, AZ, United States; ^3^Department of Neurological Surgery, Mayo Clinic, Jacksonville, FL, United States; ^4^Department of Radiation Oncology, Mayo Clinic, Rochester, MN, United States

**Keywords:** postoperative, radiosurgery, metastasis, resection, radiation

## Abstract

During the past decade, tumor bed stereotactic radiosurgery (SRS) after surgical resection has been increasingly utilized in the management of brain metastases. SRS has risen as an alternative to adjuvant whole brain radiation therapy (WBRT), which has been shown in several studies to be associated with increased neurotoxicity. Multiple recent articles have shown favorable local control rates compared to those of WBRT. Specifically, improvements in local control can be achieved by adding a 2 mm margin around the resection cavity. Risk factors that have been established as increasing the risk of local recurrence after resection include: subtotal resection, larger treatment volume, lower margin dose, and a long delay between surgery and SRS (>3 weeks). Moreover, consensus among experts in the field have established the importance of (a) fusion of the pre-operative magnetic resonance imaging scan to aid in volume delineation (b) contouring the entire surgical tract and (c) expanding the target to include possible microscopic disease that may extend to meningeal or venous sinus territory. These strategies can minimize the risks of symptomatic radiation-induced injury and leptomeningeal dissemination after postoperative SRS. Emerging data has arisen suggesting that multifraction postoperative SRS, or alternatively, preoperative SRS could provide decreased rates of radiation necrosis and leptomeningeal disease. Future prospective randomized clinical trials comparing outcomes between these techniques are necessary in order to improve outcomes in these patients.

## Introduction

While postoperative whole-brain radiation therapy (WBRT) can minimize the likelihood of both local recurrence within the surgical cavity and distant recurrence elsewhere in the brain, it has been associated with increased morbidity (1) [level 1 evidence]. WBRT can cause a clinically significant decrease in neurocognitive function (1, 2) and also quality of life (3).

Because of the increased neurotoxicity associated with WBRT, stereotactic radiosurgery (SRS) to the resected cavity has established itself as an effective alternative in the management of brain metastases after surgery. Favorable local control rates have previously been reported (4–6). Nonetheless, it is imperative to understand the factors that affect local control, patterns of failure, and symptomatic radiation-induced injury when considering SRS to the resected cavity. Among these key parameters include: appropriate target delineation (4, 6), cavity volume, margin dose and isodose selection (7), SRS timing after surgery (7), and radiologic follow-up (4). Furthermore, alongside with the adoption of postoperative SRS for brain metastases during the last decade, alternative strategies have also been developed that could minimize symptomatic radiation-induced injury and leptomeningeal dissemination. These include: multi-fractional postoperative SRS (8, 9) and preoperative SRS (10).

Given the rapidly changing literature and advances associated with the field of brain metastasis treatment, this brief review will provide a global overview of the current paradigms in the postoperative SRS applications, and outline future directions which may improve the outcome for this particular group of patients.

### Historical role of postoperative radiation for brain metastases

Patchell et al. was among the first to confirm that surgical resection of a single brain metastasis followed by whole brain radiotherapy (WBRT) improved survival when compared to patients who underwent WBRT alone (median survival 40 weeks with resection + WBRT vs. 15 weeks with WBRT alone, *p* < 0.01) (11) [level 1 evidence]. In a separate prospective randomized trial for patients with single brain metastasis, Patchell et al. subsequently found that adjuvant WBRT after resection improved local tumor control and decreased the likelihood of distant brain failure and neurologic death, compared to surgery alone (12). As a result, WBRT became an important therapeutic option in the postoperative management of patients with cerebral metastases.

Some investigators have combined WBRT and SRS postoperatively with the goal of maximizing the tumoricidal dose to the resected cavity (13, 14). For example, Roberge et al. retrospectively reviewed the outcomes of 27 patients treated with WBRT and postoperative SRS (14). Given that only one patient (4%) required surgical intervention for symptomatic radiation necrosis, the authors concluded that WBRT and SRS can be safely combined (14).

The utilization of WBRT has declined during the last decade due to increasing concerns about radiotherapy-related neurologic toxicities leading to cognitive impairment. For example, N0574 enrolled 213 patients with 1–3 brain metastases which were randomized to SRS with or with WBRT (30 Gy in 12 fractions) (15) [level 1 evidence]. The primary endpoint was cognitive function and patients treated with WBRT were 45% more likely to experience cognitive deterioration at 3 months than those treated with SRS alone. Quality of life was higher at 3 months with SRS alone, including overall quality of life and there was no difference in overall survival between the treatment groups (median overall survival 10.4 months SRS alone and 7.4 months SRS plus WBRT). These results confirmed the cognitive impact of WBRT and suggested for patients with 1–3 brain metastases amenable to radiosurgery, SRS alone was the preferred strategy.

More recently, SRS has been utilized in patients with up to 10 brain metastases. Specifically, Yamamoto et al. reported in their series that SRS without WBRT in patients with five to 10 brain metastases conferred non-inferior survival when compared to those patients found to have two to four brain metastases (16).

### Emergence of SRS for resected brain metastases

Given the neurotoxicity concerns associated with WBRT, technological improvement has been made over the last 10–15 years for utilizing SRS to the resected cavity. Multiple studies have found 12-month crude local control rates at 70–100% (4, 6, 8, 17–27), although most have been retrospective in nature. Brennan et al. reported the first prospective study on the efficacy of adjuvant SRS in patients with a limited number of brain metastases following surgery (27). Their median follow-up was 12.0 months (range: 1.0–94.1 months). Following surgical resection, 39 patients with 40 lesions were treated with SRS to the surgical bed to a median dose of 18 Gy (median time to SRS was 31 days). Their findings were consistent with a local control rate approximating 85%. Additionally, they found that non-small cell lung cancer (NSCLC) histology, tumor diameter <3 cm, and deep parenchymal tumors were associated with improved local control. Superficial dural/pial involvement and tumor diameter >3 cm were associated with increased local failure. Infratentorial lesions were at significantly increased risks of developing regional failure as opposed to supratentorial lesions.

The importance of larger target volume size (>3 cm) negatively impacting outcomes, as evidenced by Brennan et al.'s findings, has also been corroborated by other investigators as well (27). Jensen et al. reported a series of 106 patients (112 lesions) with no prior WBRT, who were treated using radiosurgery directed to the resected cavity (18). Overall survival at 12 months was 46.8%, and local control at 12 months was 80.3%. Multivariate analysis revealed that preoperative lesion diameters of >3 cm were predictive of increased local failure. Similarly, Hartford et al. reported the outcomes of 47 patients with 49 brain metastases treated with resection and postoperative SRS (26). After a median follow-up of 9.3 months, they found a 12-month local control rate of 85.5%. On univariate analysis, tumor size ≥3 cm was associated with a shorter period of time to local failure. Finally, Jagannathan et al. studied 47 patients who underwent SRS to the postoperative resection cavity following gross-total resection of the tumor (28). The mean volume of the cavity was 10.5 cm^3^. Three patients had recurrences within the resection cavity (6%). Increased surgical cavity size was associated with increased risk of local recurrence. Specifically, the volumes of these three patients' resection cavities were: 15.5, 18.4, and 21.1 cm^3^, while the mean volume for the rest of cases was 9.9 cm^3^ (28).

Degree of symmetrical expansion of the target volume for treatment has also been shown to play a key role in impacting treatment outcome. For example, Soltys et al. studied 72 patients treated from 1998 to 2006 who had SRS delivered to the resection cavity, with a median marginal dose of 18.6 Gy (6). The actuarial rate of local control at 12 months was 79%. Interestingly, improved local control was found in those treatment plans with less conformality. In fact, the conformity index was the only parameter that was significantly associated with improved local control. With this in mind, Choi et al. retrospectively studied whether adding a margin would affect treatment outcome (4). The addition of a 2-mm margin was correlated with a statistically significant reduction in local failure at 12 months from 16% to 3%, without statistically increasing clinical toxicity profiles.

Surveillance imaging is also important following SRS. In their series, Choi et al. also illustrated the importance of close follow-up and surveillance if SRS was chosen as adjuvant treatment after surgery (4). This is evident by the high rate of distant brain failure found in this series (54% at 12 months), as well as in other clinical series for surgical cavity-based SRS (recurrence rates at 44–72%) (6, 19–21, 28). Therefore, frequent surveillance imaging (typically initial follow up MRI brain 2–3 months after SRS) is strongly recommended (17).

Dose selection for resected cavity SRS has largely been dependent on the size of the postsurgical cavities on thin-slice MRI and planning CT scan. Many of the published series still utilized the SRS dose-escalation algorithm for intact tumors as outlined by the landmark trial, Radiation Therapy Oncology Group (RTOG) 90–05, and extrapolate that to the postsurgical cavity cases (27, 29). As an example, Brennan et al.'s selection of postoperative SRS doses (27) was related to the maximal surgical cavity diameter (msc) as seen from the fusion of the MRI brain and planning CT, which was: (a) 22 Gy for msc:≤2.0 cm; 18 Gy for 2.1–3.0 cm, and 15 Gy for 3.1–4.0 cm. This strategy, of margin dose reduction for large targets, has been similarly applied to the postoperative setting, including in prospective trials, to reduce the volume of normal brain exposed to high doses.

The timeliness of delivering SRS after surgery has also been highlighted by recent published studies (30, 31). For instance, Iorio-Morin et al. found that, on multivariate analysis, one of the risk factors for local recurrence included a longer surgery-to-SRS delay (more than 3 weeks) (7). Their recommendation was that SRS should take place as promptly as possible, with a target date of 3 weeks after surgical resection. These recommendations are consistent with Patel et al. who recently postulated, based on his findings of an increase in the tumor bed cavity size after surgery (32), that delaying postoperative SRS beyond 3 weeks in hopes of significant tumor bed cavity contraction should not be advised. Performing SRS within 2–3 weeks after surgery may be the ideal balance for allowing the patient to recover surgically, without excessive delay in postoperative treatment that could increase the risk of tumor recurrence.

### Expert consensus on accurate contour delineation in tumor bed radiosurgery

Recently, Soliman et al. published their consensus guidelines on accurate contour delineation in tumor bed radiosurgery (33). This is the first study published which comprehensively provides guidelines on design for the appropriate treatment SRS volumes for resected cavity cases. Here, internationally recognized authorities in the field each contoured ten postoperative completely resected cases of diverse clinical scenarios and cases consisted of tumors located in various regions of the brain. The level of agreement was adequate (mean sensitivity and specificity were 0.75 and 0.98, respectively). There were two cases of metastatic disease in the infratentorial compartment where significant differences were detected among the contours, in regard to how generous the clinical target volume (CTV) should be along the bone flap.

This finding led the researchers to propose the following recommendations in regard to CTV design. First, the CTV should completely cover the contrast-enhancing surgical cavity with the use of the T1-weighted gadolinium-enhanced axial MRI scan, excluding any vasogenic edema; second, the CTV should completely encompass the surgical tract visualized on postoperative imaging; third, if preoperatively there was tumor contacting the dura, the CTV should include a 5–10 mm margin along the bone flap that extends beyond the area where there was existing contact before surgery; fourth, if there is no contact that is identified between the tumor and dura, the CTV should include a margin of 1–5 mm where the bone flap is located; finally, if there is any contact pre-operatively with any of the venous sinuses, there should be a 1–5 mm margin applied to the CTV in the area where the sinus is located (33). Hence, the authors concluded that venous sinus/meningeal coverage should be generous in the CTV to prevent failures in these high risk regions. While this study is helpful, it is important to recognize that these recommendations are based on expert opinion and further study is needed.

### Complications and recurrence patterns following postoperative SRS

Radiation necrosis is a known potential complication after SRS and can be difficult to distinguish clinically and radiographically from tumor progression (34). Advanced techniques for distinguishing radiation necrosis from tumor recurrence now include nMR spectroscopy using Choline/N-Acetyl Aspartate and Choline/Creatine ratios and MR perfusion with the use of relative cerebral blood volume (35, 36). Current treatment options include glucocorticoids, hyperbaric oxygen, and surgery, bevacizumab, and focused interstitial laser thermal therapy, with varying degrees of effectiveness (37).

The literature has shown rates of radiographic radiation necrosis in patients treated with SRS (for intact brain metastases) to be as high as 24% (34). Meanwhile, wider ranges of rates of radiation necrosis have been observed after postoperative SRS. These rates of radionecrosis in tumor bed SRS cases have ranged from 1.5 to 18.5% (9, 27, 38–40). As reported by Keller et al., the infratentorial location was predictive of increased radionecrosis (hazard ratio [HR]: 2.97; 95% confidence interval [95% CI]: 1.47–6.01; *p* = 0.0025) (40). The V14Gy (volume of brain receiving 14 Gy) was also associated with the risk of radionecrosis following resected cavity SRS.

Regarding tumor progression, elsewhere intraparenchymal tumor progression remains the predominate location of intracranial failure after SRS for brain metastases. While these recurrence rates are generally similar to patients receiving SRS alone for intact brain metastases, some have postulated that rate of leptomeningeal carcinomatosis is higher with resection of brain metastases and surgical violation of the tumor capsule (30). The overall rate of leptomeningeal disease (LMD) in solid malignancies is estimated in the range of 5–15%, and varies based on several clinical and pathologic features (17, 30, 31, 41), and LMD rates followed resected cavity SRS have ranged from 8 to 24% (18, 20, 42). Current research has yet to pinpoint whether LMD in the postoperative SRS setting is a manifestation of the tumor's natural history, or if dissemination is secondary to the surgery itself (tumor spill) and whether postoperative SRS may also be indirectly contributing to it (higher incidences of distant tumor progression leading to LMD, etc.) (9). Several series have reported on this topic; for example, Atalar et al. reported a higher rate of LMD in patients with metastatic breast cancer which may be related to tumor biology (43). It has also been shown that piecemeal resections and brain metastases in the infratentorial compartment may lead to increased LMD (17, 20, 44). While tumor histology has clear implications on systemic therapy options and patient survival, there are very limited data to support a differing postoperative radiosurgical management (dose, timing, etc.) based on tumor histology alone.

### Alternative strategies: fractionated and preoperative SRS

**Figure 1:**
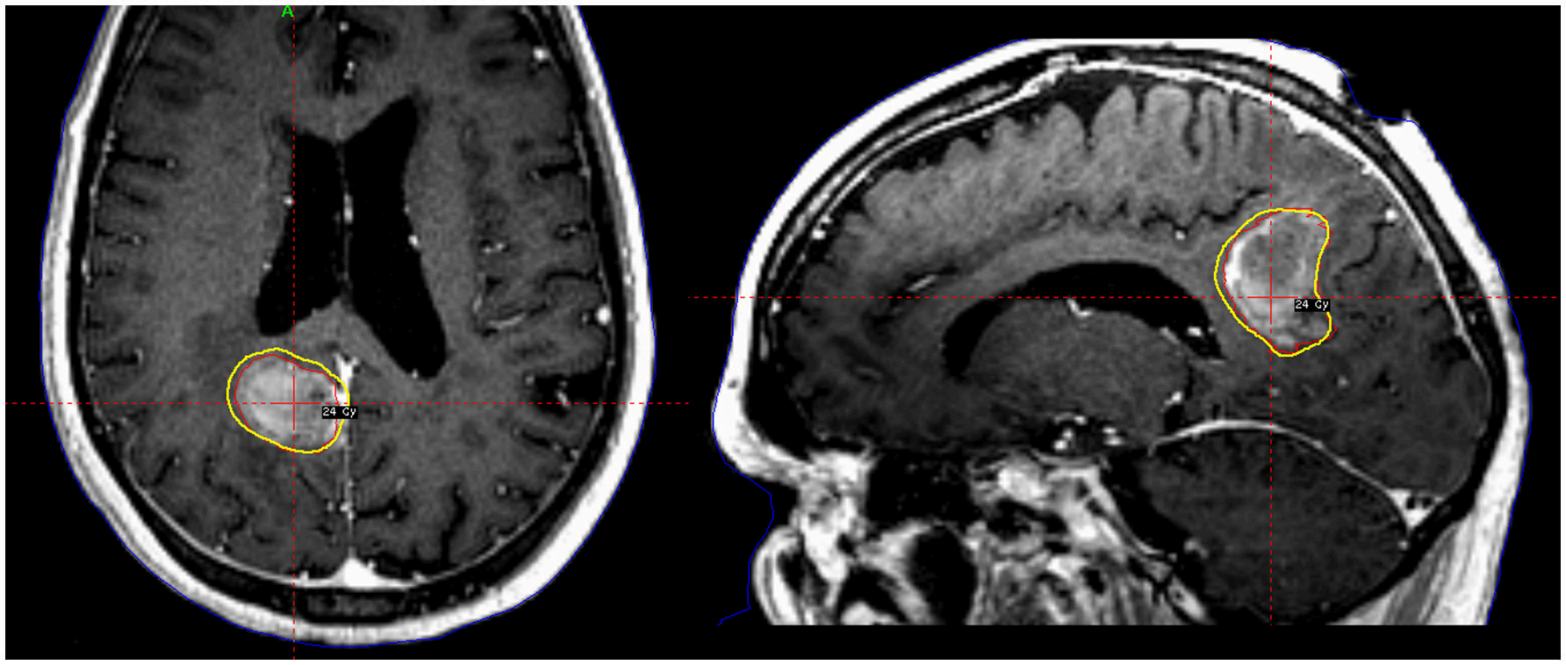
A patient with a large tumor cavity following resection for brain metastasis received postoperative fractionated stereotactic radiosurgery to 24 Gy in 3 fractions.

Single-fraction SRS may have increased side effects, particularly for lesions >3 cm (27), or those located in eloquent regions (45, 46). As a result, hypofractionated SRS is a reasonable alternative to single-fraction SRS in both preserving tumor control and also reducing radionecrosis (Figure 1). Steinmann et al. studied 33 patients with single brain metastasis (median volume, 25.6 cc) who underwent surgery followed by hypofractionated SRS (8). A high local control rate of 71% at 1 year was achieved. Similarly, Wang reported a local control of 80% at 6 months with hypofractionated treatments. Meanwhile, Keller et al. published a series of 181 patients treated with a 3-fractionation schedule of 33 Gy to the resected cavity. The 1-year local control rate was 88% (9). Interestingly, on multivariate analysis, tumor contact with the meninges was predictive of increased local failure, which validated the importance of adding generous margin to the CTV in that region as suggested by the consensus guidelines as discussed above (33).

**Figure 2:**
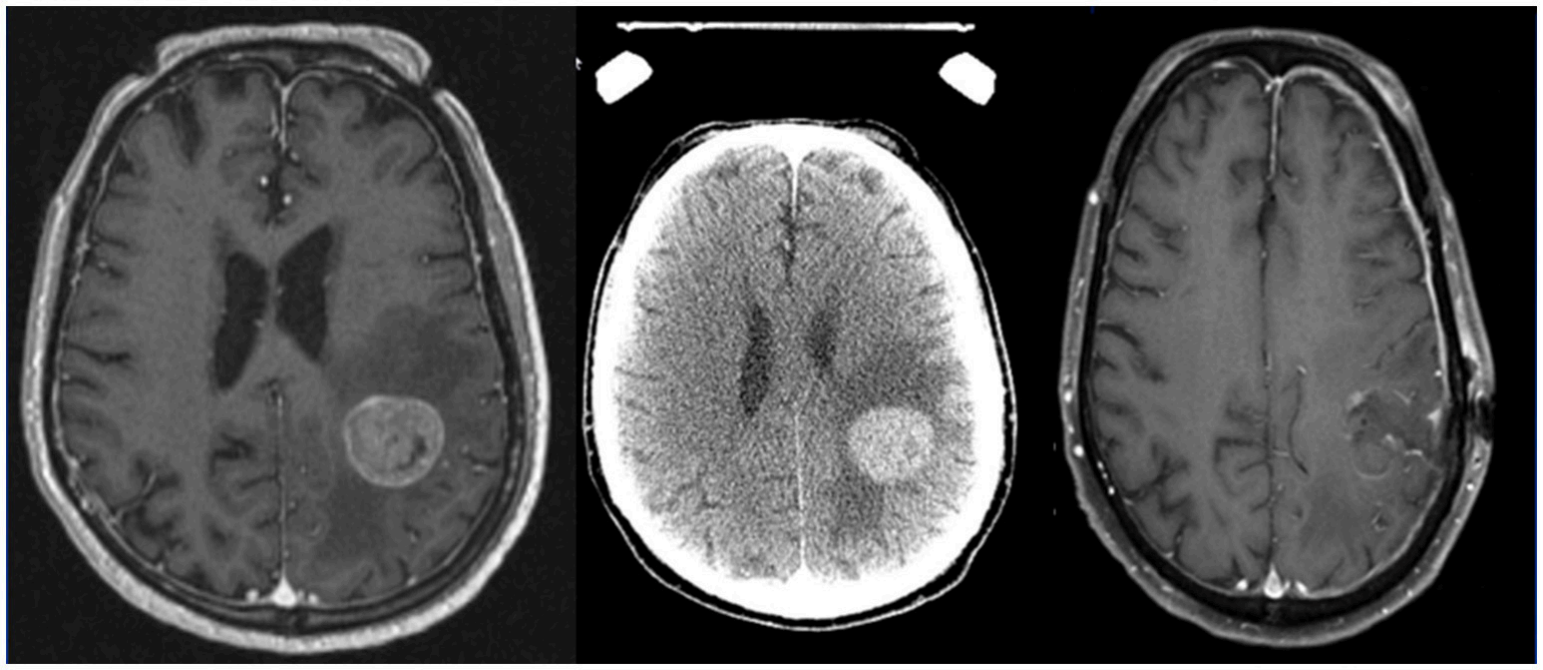
Axial MRI and CT images of a patient with a brain metastases treated with preoperative stereotactic radiosurgery followed by resection days later.

**Figure 3:**
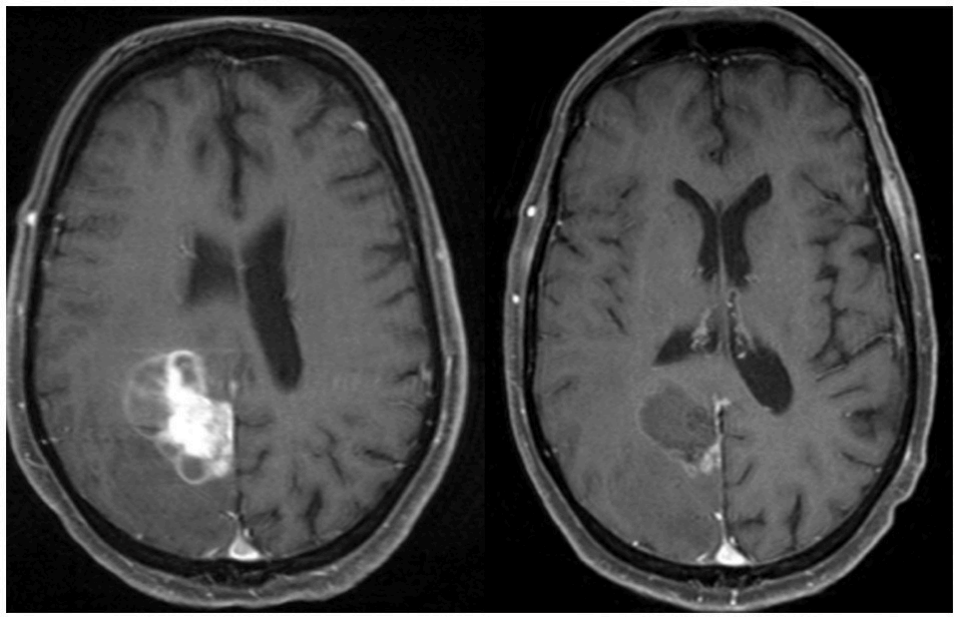
Axial MRI and CT images of a patient with a brain metastases treated with preoperative stereotactic radiosurgery followed by resection days later.

Preoperative SRS is another potential strategy (Figures 2, 3). Recently, Patel et al. published findings from a multi-institutional study retrospectively comparing outcomes in the pre-operative vs. postoperative SRS settings (66 and 114 patients, respectively) (10). With a median follow-up of 24.6 months, no difference was found between groups for overall survival, local recurrence, or distant brain failure. However, surprisingly, postoperative SRS had significantly increased rates of LMD and symptomatic radionecrosis. A follow-up study was subsequently performed comparing outcomes between preoperative SRS and postoperative WBRT (47). A total of 102 patients were analyzed (66 in the pre-SRS group, vs. 36 in the post-op WBRT group), and the authors reported the 12-month overall survival rates were similar between groups, as were 24-month outcomes for local control, distant control, and the presence of LMD. Crude rates of radiation necrosis were 5.6 and 0% for the preoperative SRS and the postoperative WBRT groups, respectively. Future prospective studies should direct their effort to address whether preoperative SRS may be superior to postoperative SRS, and to evaluate the optimal radiation doses, timing between surgery and SRS treatments, and also salvage options for these patients requiring locoregional control for their limited brain metastases (9).

### Recent prospective studies

In the past year, two randomized prospective trials have been published highlighting the effectiveness of postoperative SRS. Mahajan et al. compared 2 groups: those who underwent SRS (64 patients) vs. those who were observed after gross total tumor resection (68 patients) (48) [level 1 evidence]. Findings revealed that the 12 month freedom from local recurrence was significantly higher in the SRS group (72% in the SRS group vs. 43% in the observation group). In both groups, there were no adverse events or deaths attributed to either treatment. This study validated the recommendation for adjuvant radiosurgery even after gross total neurosurgical resection.

In a parallel cooperative group study, Brown et al. enrolled 194 patients randomized between postoperative SRS (98 patients) or WBRT (96 patients) (49). The results showed a greater decline in cognitive function, worse quality of life, and worse functional independence with the use of WBRT as compared to SRS. Although surgical bed control and intracranial control were improved with WBRT, there was no difference in overall survival was observed between the two groups.

### The future of postoperative radiosurgery

Postoperative SRS is associated with an acceptable rate of local control by multiple studies, and causes less neurotoxicity when compared to WBRT. As a result, postoperative SRS should be regarded as a standard of care in lieu of WBRT after surgery. Nonetheless, while most patients will still develop distant failure after SRS treatment, the ability of postoperative SRS to spare or delay WBRT is an important advantage and of significant appreciation clinically by the patients who may otherwise require upfront WBRT.

Recent collaborative efforts such as Soliman et al. have been essential in establishing a standard on how to safely and successfully execute radiation contouring treatment design in tumor bed SRS cases (33). However, it is not yet known how to best utilize preoperative tumor extent in postoperative SRS target delineation. Moreover, margin dose selection and contouring techniques should be employed for unusual cases such as those with hemorrhage in the surgical cavity and those with piecemeal resections (33).

It remains to be seen whether prospective studies can show (a) a benefit in local control and/or improved toxicity for hypofractionated tumor bed SRS vs. single fraction tumor bed SRS and (b) whether pre-operative SRS can lead to decreased risks of radiation necrosis and/or LMD vs. postoperative SRS, and studies are underway to analyze these strategies.

In parallel, advancements in systemic therapy's intracranial effectiveness could be leveraged in combination with SRS. For example, we have witnessed the rapid rise in the use of immunotherapy as the first line therapy for many metastatic cancers (lung, melanoma, and renal). Recently, prospective data has emerged which supports the use of immunotherapy alone (without local therapy) for small, asymptomatic brain metastases. Specifically, a phase II study of the PD-1 (anti-programmed cell death protein 1) antibody pembrolizumab in patients with brain metastases from non-small cell lung cancer and melanoma (NCT02085070) was published showing that a durable brain metastasis response was achieved in 22% of patients with melanoma and 33% of patients with NSCLC (50). Toxic effects were consistent with those reported in previous trials of pembrolizumab in these diseases and neurological adverse events associated with drug or disease were infrequent and non-life threatening (50). The results of many prospective studies that combine immunotherapy and SRS are pending, which could inform synergy between these modalities to improve local, as well as distant, tumor control (51). Intraoperative radiation therapy (IORT) has also been postulated as an alternative technique to improve the results of postoperative SRS, and likewise it may also prove to be a highly efficacious technique when combined with immunotherapy (52).

There are several active areas of research that may serve to redefine the role of radiosurgery in patients with metastatic cancer, not the least of which is immuno-radiosurgery. Recently, there has been a flurry of reports of possible synergy between radiosurgery and various immunomodulatory systemic agents (51, 53). Radiosurgery will, without doubt, play a key role in the management of patients with metastatic disease in the future. As advances in surgical techniques, radiosurgical delivery, and systemic therapies develop, the relative role of these strategies will need to be continually refined.

## Author contributions

EM and DT contributed conception and design of the mini-review. EM wrote the first draft of the manuscript. DT contributed the figures enclosed in the manuscript. All authors contributed to manuscript revision, read and approved the submitted version.

### Conflict of interest statement

The authors declare that the research was conducted in the absence of any commercial or financial relationships that could be construed as a potential conflict of interest.
